# Analysis of the Composition of Lyophilisates Obtained from *Aloe arborescens* Gel of Leaves of Different Ages from Controlled Crops

**DOI:** 10.3390/molecules26113204

**Published:** 2021-05-27

**Authors:** Kamil Pawłowicz, Dominika Ludowicz, Marta Karaźniewicz-Łada, Kamil Wdowiak, Judyta Cielecka-Piontek

**Affiliations:** 1 Phytopharm Klęka S.A., Klęka 1, 63-040 Nowe Miasto nad Warta, Poland; Kamil.Pawlowicz@europlant-group.pl; 2 Department of Pharmacognosy, Faculty of Pharmacy, Poznan University of Medical Sciences, Święcickiego 4, 60-781 Poznań, Poland; dsiakowska@ump.edu.pl (D.L.); the3nigmav2@gmail.com (K.W.); 3 Department of Physical Pharmacy and Pharmacokinetics, Faculty of Pharmacy, Poznan University of Medical Sciences, Święcickiego 6, 60-781 Poznań, Poland; mkaraz@ump.edu.pl

**Keywords:** *Aloe arborescens*, aloin A, aloenin A, polyphenols, antioxidant potential

## Abstract

The aim of the study is to evaluate the composition of lyophilisates obtained from *Aloe arborescens* leaf gel at the age of one to four years. The leaves were obtained from controlled crops, which allowed to exclude environmental factors as variables. It was confirmed that the lyophilisates obtained from different years of *Aloe arborescens* leaf gel varied in chromatographic analyses in terms of aloin A and aloenin A content (high-performance liquid chromatography with diode-array detection HPLC-DAD, high-performance liquid chromatography with tandem mass spectrometric detection HPLC-MS/MS). Similarly, while testing the phenolic acids and the sum of polyphenols content, differences in their levels in leaf gel lyophilisates from plants of individual years were observed (spectrophotometric method UV-VIS). The lyophilisate composition analysis showed that the one-year-old leaves were characterized by the highest content of aloin A and aloenin A. While the content of polyphenols, including phenolic acids, was higher in the leaves of older plants. The antioxidant potential of the tested lyophilisates was assessed simultaneously. Regardless of the research model used (CUPRAC, DPPH, ABTS), an antioxidant effect was noted for *Aloe arborescens* leaves.

## 1. Introduction

Plant raw materials contain numerous groups of compounds with a biological effect. Especially the group of secondary metabolites in plant compounds show biological activity [1,2,3,4,5]. The synergism of the activity of selected metabolites shapes the final pharmacological effect of the plant material. There are many reports in the literature on the subject of the synergism of the action of various groups of secondary metabolites (e.g., *Cannabis* spp.) [6,7], also, of the concept of the so-called entourage, i.e., achieving better pharmacological parameters as a result of the presence of groups of secondary metabolites [8,9,10]. Secondary metabolites, as compounds produced as a defense reaction, respond to the variability of many environmental factors, which may modify the composition of secondary metabolites produced by the plant [11,12,13]. In the case of relatively constant external factors of perennial medicinal plant cultivation (example of controlled crops), the plant’s age is an essential factor determining its secondary metabolite composition [14]. For almost every medicinal plant, it is important to obtain the raw material in the period of the optimal composition of secondary metabolites.

*Aloe* is a large genus of significant medical, cosmetic, and food importance, consisting of, according to Salehi et al., over 446 species belonging to the *Xanthorrhoeaceae* family, of which many plants are well known for centuries for their medicinal properties [15,16]. There are differences in the morphological and anatomical features between species, as these perennials exist in the form of lianas, shrubs, and ‘trees’. However, the gel present in the leaves is characteristic for all Aloe genus plants and is considered a valuable raw material for preparing various forms of herbal drugs, dietary supplements, cosmetics, and functional food. Numerous scientific reports and clinical studies emphasize the multidirectional effect of leaf gel of all Aloe spp. plants on the human body [17,18].

The most known plants of the genus include *Aloe arborescens* Mill., *Aloe ferox* Mill., *Aloe artistata* Haw., *Aloe saponaria* Ait., *Aloe polyphylla* Schonl. ex *Pillans*., and—the most famous—*Aloe vera* L. These plants occur naturally in dry, savannah, and desert conditions in eastern and southern Africa and Madagascar. Over the centuries, Aloe has reached the Mediterranean, warm regions of Europe, Asia, and the Americas, and crops grown in these regions under controlled conditions in greenhouses also provide valuable raw material [19].

A significant species, due to the possibility of obtaining medicinal products, is *Aloe arborescens*. The rich and diverse composition of *Aloe arborescens* determines its multidirectional application in herbal medicine [20,21,22,23]. The multiple biological effects of Aloe result from the presence of several classes of biological compounds in the leaves, which are plant raw material. The leaves have different chemical constituents depending on the part—leaf peel, leaf gel [24]. As the most important groups of isolated compounds should be indicated: (i) glycoproteins: exhibit anti-insect, antitumor, immunomodulatory, antimicrobial, and antiviral properties and may inhibit HIV-1 reverse transcriptase activity, (ii) polysaccharides: 1–4 linked glucomannans, linear glucan, and branching arabinose with galactose presenting a potent phagocytosis-enhancing effect; (iii) enzymes: carboxypeptidases which reveal anti-inflammatory and anti-thermal burn action properties, (iv) phenolic compounds: (aloenin, barbaloin, aloe-emodin) may inhibit gastric juice secretion and present anti-inflammatory activity [25].

One of the effects associated with the presence of active compounds in *Aloe arborescens* is anticancer activity against liver cancer, resulting from the inhibitory effect on induction of preneoplastic glutathione S-transferase positive hepatocyte foci GST-P + − colon cancer (duodenal, intestinal cancer) [26]. Picchietti et al. confirmed the immunomodulatory effect of *A. arborescens* in bacterial and viral infections [25]. In comparison, Mogale et al. investigated the antidiabetic activity of aqueous extract of *A. arborescens* leaf gel based on the protection mechanism of pancreatic β-cells [27]. The antioxidant and anti-inflammatory activity should also be pointed out in addition to the mentioned properties of *Aloe arborescens* [28]. Moreover, similarly to other species of the genus, *Aloe arborescens* is characterized as a strong laxative agent [29].

A comprehensive analysis of selected Aloe plants carried out by Zapata et al. enables the comparison of *Aloe arborescens* with other species of the genus. It is characterized by the highest total phenolic concentration and total antioxidant activity. This research team also conducted an analysis of the content of active compounds in *Aloe arborescens* leaves depending on the harvesting period—the comparison concerned leaves harvested in spring, summer, and winter. In all cases, the highest content was found for leaves harvested in summer [30].

So far, no relationship has been reported between the age of *Aloe arborescens* (one-, two-, three-, and four-year-old plants) and the biological properties of groups of compounds contained in leaves obtained from plants of subsequent years. Therefore, the aim of this study is to evaluate the composition of one-, two-, three-, and four-year-old *Aloe arborescens* leaves. *Aloe arborescens* leaves that were collected from controlled crops are used as the material for the study.

## 2. Results

**Figure 1:**
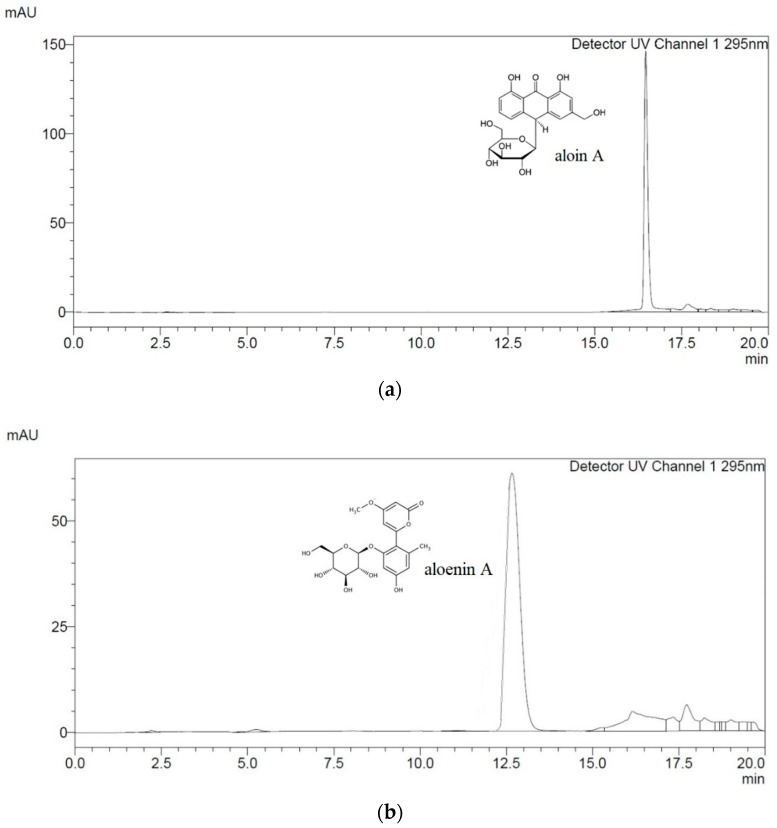

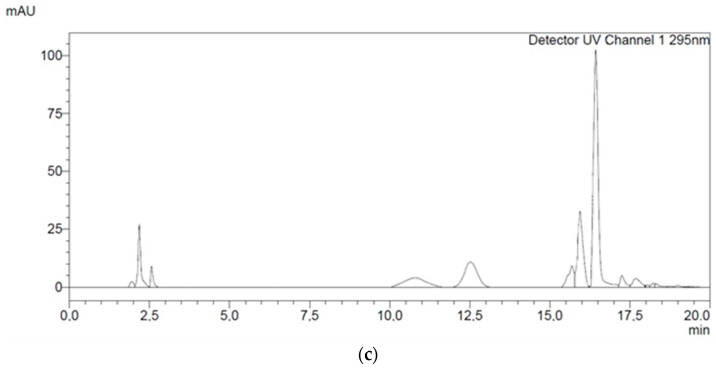
The HPLC (high-performance liquid chromatography) chromatograms of analytical standards and lyophilisate: (**a**) aloin A; (**b**) aloenin A; (**c**) *A. arborescens* lyophilisate extract.

Bearing in mind the need to standardize the raw material from *Aloe* spp., in our research, at first, the contents of aloin A and aloenin A were determined and compared in lyophilisates obtained from *Aloe arborescens* leaves of the years one to four. For this purpose, the high-performance liquid chromatography with diode-array detection (HPLC-DAD) method with the detection wavelength λ_max_ = 295 nm and the mobile phase comprised of methanol and water in a gradient was developed and validated (Table S1, Supplementary Materials). Under the developed chromatographic separation conditions, the following were eluted: aloenin A (R_T_ = 12.7 min) and aloin A (R_T_ =16.5 min) (Figure 1).

**Figure 2:**
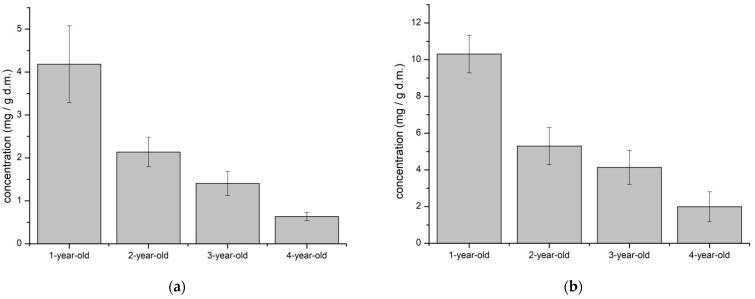
The content of standard substances in studied ethanol extracts: aloin A (**a**); aloenin A (**b**).

On the basis of the calibration curves for the reference substances, quantitative analysis was possible (Figure 2). The aloin A content was highest in one-year-old leaves. Similarly, aloenin A also was found in the highest content in one-year-old leaves. It can be noticed that the content of aloin A and aloenin A decreased with the aging of the plant.

**Figure 3:**
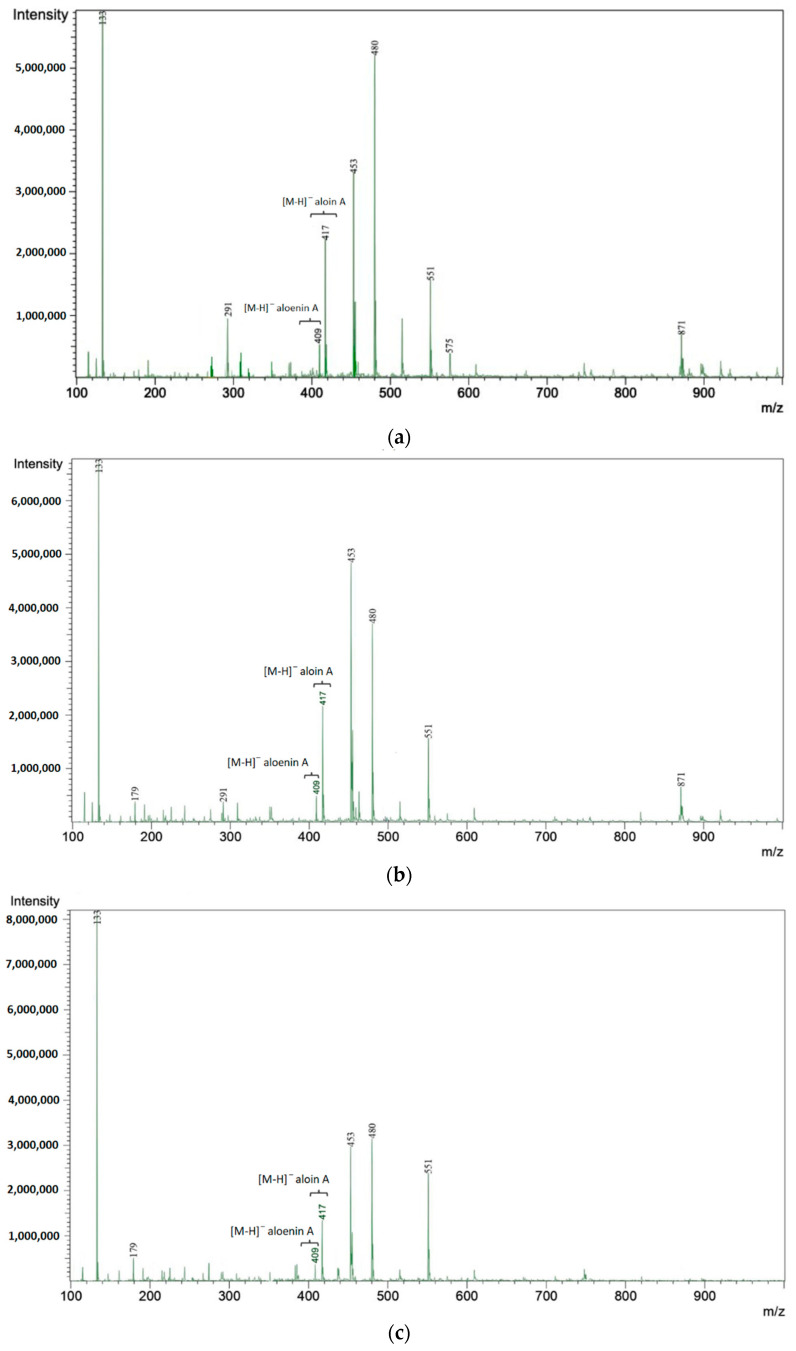

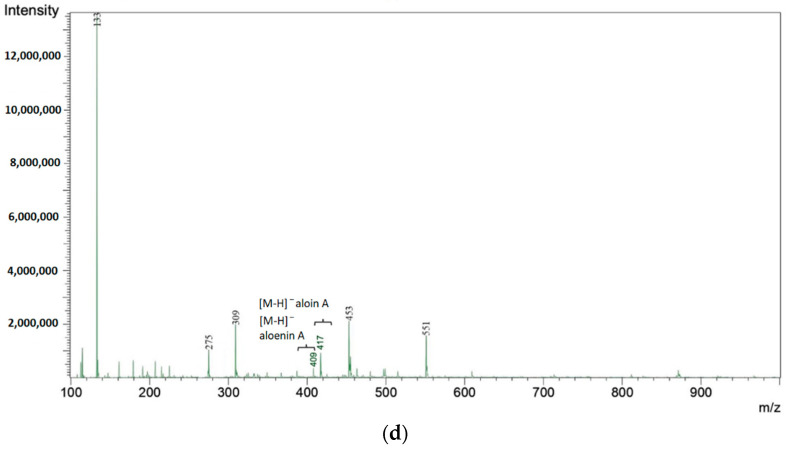
Mass spectra for studied extracts of one-year-old (**a**); two-year-old (**b**); three-year-old (**c**) four-year-old (**d**) leaves.

The high-performance liquid chromatography with tandem mass spectrometric detection (HPLC-MS/MS) analysis was performed to confirm the presence of selected active compounds in the studied extracts (Figure 3). Mass spectrum of aloin A showed [M-H]^−^ at *m*/*z* 417 and a daughter ion at *m*/*z* 297 (Figure S1, Supplementary Materials), which resulted from the loss of [C_4_H_8_O_4_]^−^. Aloenin A displayed [M-H]^−^ at *m*/*z* 409 and daughter ions at 247 (corresponding to the loss of [C_6_H_10_O_5_]^−^) and 171 (Figure S2, Supplementary Materials). Moreover, a high concentration of fragment ions of unknown origin at *m*/*z* 239, 242, and 266 in the positive ionization mode and at *m*/*z* 453 and 480 in the negative ionization mode was noticed. These came from polyphenolic structures, and their sum differed for lyophilisates from individual years. Examples of such compounds include condensates with caffeic acid, whose characteristic base peak, visible in Figure 3, is at *m*/*z* 179. Differences in intensities of the discriminative ions may be caused by the aging of the plant.

**Figure 4:**
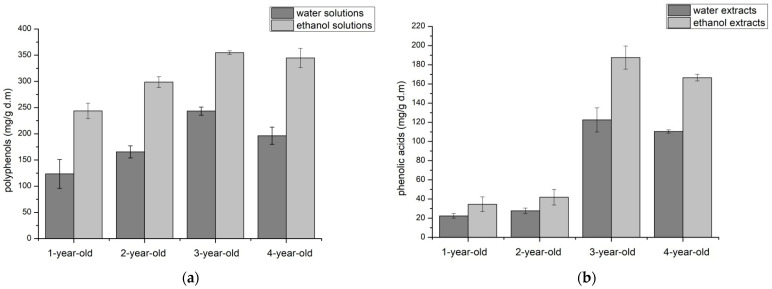
Concentration of (**a**) polyphenols and (**b**) phenolic acids in studied extracts.

As the next stage, the content of phenolic compounds in *Aloe arborescens* leaf lyophilisates obtained from controlled crops for one-, two-, three-, and four-year-old plants was assessed. Obtained results showed the highest content of polyphenols for three-year-old plants. In one- to three-year-olds, the content in the tested extracts increased, and for four-year-old plants, the gel lyophilisates were lower than for three-year-old plants (Figure 4a). When it came to phenolic acids content analysis, lyophilisates from three-year-old leaves had by far the highest content of this group of compounds, and it was much higher compared to leaves from other years (Figure 4b).

**Figure 5:**
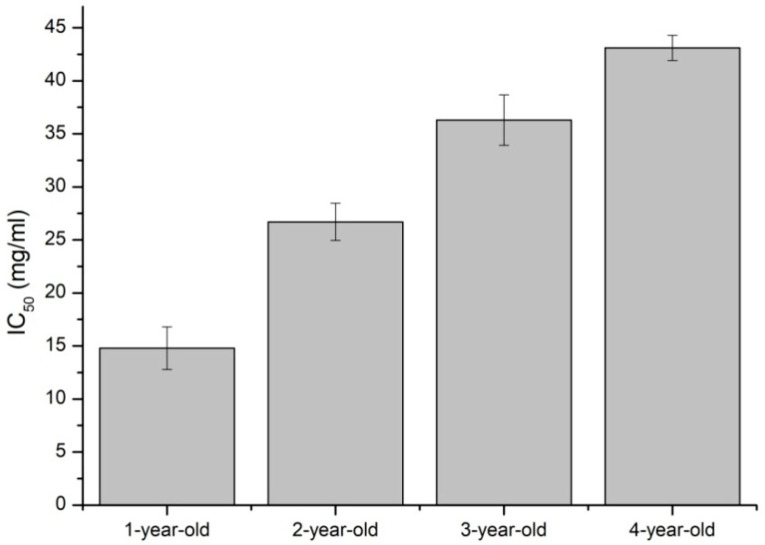
IC_50_ values for studied ethanol extracts obtained in studying antioxidant properties by CUPRAC assay.

All of the tested leaves showed antioxidant properties. During the CUPRAC assay, the determined IC50 values for the examined leaves were between 14.8–43.1 mg/mL. The lowest concentration needed to inhibit the complex of neocuproine’s oxidizing properties and copper ion (II) exhibited in the ethanol extract prepared from the lyophilized gel from the leaves of the one-year-old plant. The IC50 values were higher for ethanol extracts of two- and three-year-olds, and the weakest antioxidative properties characterized the four-year-old *Aloe arborescens* extract (Figure 5). The aqueous extracts showed such weak antioxidant properties that it was impossible to determine the IC50 values for three- and four-year-old plants. Therefore the results are not included in the figure.

**Figure 6:**
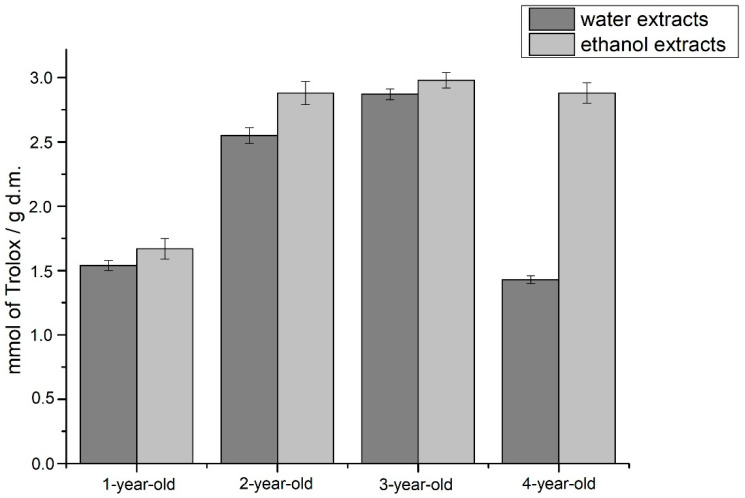
Results of antioxidant potential of studied extracts obtained with the use of DPPH radical.

The results of the next applied antioxidant assay—DPPH method—indicated three-year-old aloe leaves as the ones with the most potent antioxidant properties, slightly weaker were the properties of two-year-old plants, then four-year-old plants, and the weakest antioxidant properties in the test using the DPPH radical showed in the extracts obtained from one-year-old plants (Figure 6).

**Figure 7:**
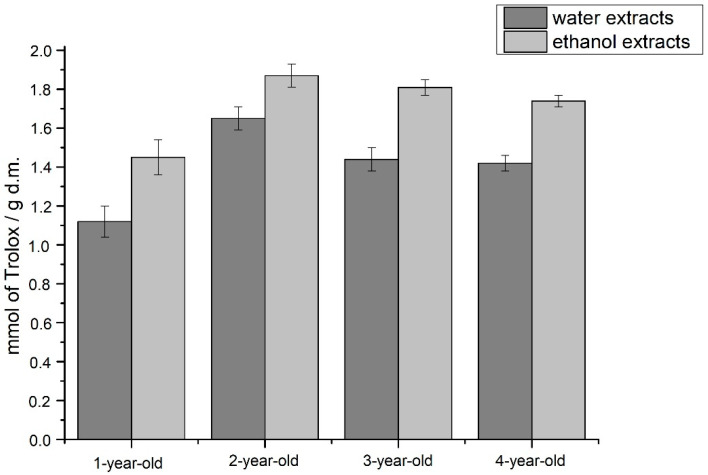
Results of antioxidant potential of studied extracts obtained with the use of ABTS radical.

In the study of the antioxidant properties using the ABTS radical, the most potent antioxidant properties were observed for the studied extract of two-year-old plants, weaker for three- and four-year-old, and the weakest for one-year-old plants (Figure 7).

Statistical analysis of the results of antioxidant studies and investigations of phenolic content revealed statistically significant differences in all assays (Table S3, Supplementary Materials).

## 3. Discussion

Based on literature reports on the differences in the chemical composition of *Aloe* leaves depending on the harvesting season, *A. arborescens* leaves collected in late summer were selected as the subject studied [30]. Zapata et al. conducted the research for various *Aloe* spp. (*A. arborescens*, *A. aistata*, *A. claviflora*, *A. ferox*, *A. mitriformis*, *A. saponoaria*, *A. striata*, *A. vera*), which resulted in information on the season’s influence (spring, summer, winter) on the content of aloin, free putrescine, spermidine, and phenolic compound, and hydrophilic and lipophilic total antioxidant activity. For the species *A. arborescens*, the highest concentrations of tested compounds were noted for leaves harvested in summer [30].

Our research aimed to examine the relationship between the age of leaves (one-, two-, three-, four-year-old) of *A. arborescens* and the contents of specific compounds (aloin A, aloenin A, polyphenols, and phenolic acids) in gel lyophilisates. There are reports in the literature on the therapeutic multidirectional beneficial effects of the *Aloe* spp. on various body systems: gastrointestinal, respiratory tract, central and peripheral nervous system, skin, and eyes [16,31].

*A. arborescens* controlled greenhouse crops are an excellent model for comparing mainly the leaf content variation, due to the plants’ age. Other variables such as soil, hydration, temperature, air humidity, and light exposure become eliminated and are considered constant parameters. As far as the harvesting cycle is concerned, in the case of Phytopharm Klęka crops, the age of the plant is counted from planting the seedling to the ground. In turn, as pharmaceutical raw material, the three-year-old and older leaves are used. In our research, for extract preparation, we used lyophilisates from one-, two-, three-, and four-year-old gel of leaves of *A. arborescens*. The use and storage of the lyophilisates avoided variations in the content of compounds, resulting from the easy evaporation of solvents commonly used in the preparation of extracts. Freeze-drying and extraction processes can be indicated as stages of storage and preparation of the raw material that does not change its quality.

As a result of the conducted research comparing the composition of lyophilisates obtained from the gel of the leaves of different ages, it was found, using the HPLC-DAD method, that the differences in the levels of aloin A were large and amounted to over 50% between individual years. The laxative effect based on hyperemia of pelvic organs may be associated with aloin A content, which in the large intestine under the influence of enzymes—glycosidases—releases the aglycone (aloe-emodin) [32]. Therefore, one-year-old leaves are considered the best plant material for the production of products with this indication.

The trend of the aloenin A level was similar—the highest in one-year-old plants. Aloenin A is not a compound found in all species of the genus *Aloe*. Its presence has been confirmed in 16 *Aloe* species out of 380 tested. The detection of aloenin has always been associated with the presence of aloin [33]. Taking into account the antioxidant properties of aloenin A and its potential in the stimulation of hair growth, as well as the beneficial effects of *Aloe* on the skin, one-year-old *A. arborescens* leaves seem to be the best candidate for testing the possibility of use in skin diseases from the studied years [34,35,36]. Additionally, due to the low-molecular structure, stability, and relatively high content of *Aloe* gel, aloenin A seems to be an excellent marker for the standardization of *Aloe arborescens* preparations [37].

All of the tested lyophilisates are characterized by antioxidant activity. The compounds present in the lyophilisates have the potential to neutralize free radicals. Polyphenols are biologically active compounds that neutralize free radicals by donating an electron or hydrogen proton, metal chelation, induction of antioxidant enzymes, and action as chain breaker of lipid peroxidation [38]. The polyphenols group includes various groups of compounds that contain a phenolic structure [39]. One of them is a group of phenolic acids. The properties of phenolic acids are well documented with respect to their protective health effects, such as antimicrobial, anticancer, anti-inflammatory, and anti-mutagenic properties [40].

Using the CUPRAC technique, the highest antioxidant potential for lyophilisates was obtained for one-year-old leaves. The analysis of the composition showed that in these lyophilisates, the levels of aloin A and aloenin A (4.18 and 10.31 mg/g d.m., respectively) are the highest, which is 2–3 times more than the lyophilisates obtained from the leaves of older plants. However, it showed the lowest quantity of polyphenols and phenolic acids, so it can be concluded that antioxidant activity presented by lyophilisates in CUPRAC assay is not affected by the presence of these groups of compounds. Therefore, in this case, the antioxidant property of extracts can be connected to the existence of other classes of compounds such as anthraquinones, e.g., aloin A, which are proven to be present in the extract or other groups which were not studied in our case. Moreover, there can be seen a relationship between the content of anthraquinones and CUPRAC assay results; with a decreasing amount of anthraquinones in the extract, there was a drop in the antioxidant potential of lyophilisates. This may suggest that anthraquinones, in the case of *Aloe arborescens* extracts, are responsible for the antioxidant effect by cupric ion reducing activity. This observation is in line with other studies, where it was confirmed that anthraquinones possess antioxidant activity via radical scavenging effect and ion reducing activity [41,42,43].

The potential to act against the oxidation process was also determined by the DPPH technique. The study showed that the lyophilisates obtained from three-year-old leaves have the highest antioxidant potential, which may be associated with a high content of polyphenols (354.88 mg/g d.m.) and phenolic acids (187.43 mg/g d.m.). Higher antioxidant potentials were obtained for ethanol extracts, and the difference was particularly large for extracts obtained from lyophilisates of four-year-old leaves.

The last technique for investigating the antioxidant potential was the ABTS method. As in the DPPH technique, ethanol extracts showed the greater potential, regardless of the leaves’ age from which the gel to obtain the lyophilisates came. The highest antioxidant properties were characteristic for the lyophilisates for two- and three-year-old plants. In this case, similar to the DPPH method, taking into account the content of phenolic compounds, it can be suggested that the antioxidant activity is also due to the presence of other compounds, e.g., a synergy between the action of polyphenols and anthranoid compounds can be suggested. Moreover, according to the literature on the subject, it can be seen that the choice of the alcoholic extraction solvent may affect the antioxidant activity of the extract—an example is the use of ethanol which causes the transition to the extract of more compounds with antioxidant activity potential [44,45].

Due to the potent antioxidant properties of polyphenols, including phenolic acids, it could be expected that their high content in three-year-old leaves would translate into their potentially highest antioxidant activity. However, this relationship can only be applied to the DPPH assay, where extracts from three-year-old leaves showed the best antioxidant activity, which can be connected to the highest amount of polyphenols and phenolic acids present in the raw material. This connection is especially seen in water extracts, where the one from three-year-old leaves revealed a higher quantity of polyphenols and a significantly higher amount of phenolic acids with respect to water extracts obtained from leaves in different ages. The methods used to test the extracts’ antioxidant activity indicated that the one-, two-, and three-year-old leaves showed the highest activity, depending on the type of study (CUPRAC, DPPH, ABTS method, respectively). Therefore, it can be concluded that, besides phenolic acids, other compounds from the polyphenol group and other groups will affect the antioxidant properties.

The results of the study may indicate the beneficial usage of one-year-old and two-year-old leaves of *Aloe arborescens* as a pharmaceutical material, since they are characterized by different quality and quantity properties, especially when it comes to anthranoid compounds composition. It could be advantageous to use the early age leaves as a material in the development of laxative agents.

## 4. Materials and Methods

### 4.1. Plant Material

**Figure 8:**
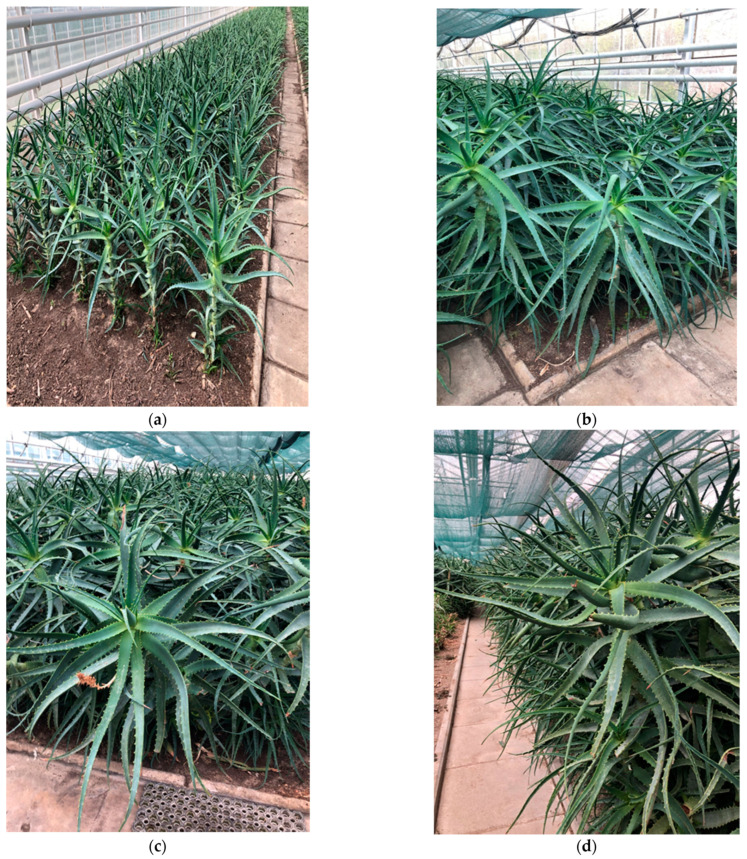
*Aloe arborescens* crops in Phytopharm Klęka. (**a**) One-year-old plants; (**b**) two-year-old plants; (**c**) three-year-old plants; (**d**) four-year-old plants.

*Aloe arborescens* Mill. fresh leaves supplied by Phytopharm Klęka S.A. (Klęka, Poland) were used for the study. The leaves were obtained from the one-, two-, three-, and four-year-old plants growing in controlled crops (Figure 8).

### 4.2. Crop Conditions

Cultivation of *Aloe arborescens* Mill. was carried out in controlled, inbreed plantations in heated greenhouses. Depending on the season, temperature varies between 10 °C to 30 °C as plants do not withstand temperatures below 0 °C. During cultivation, ventilation and shading are applied in order to regulate temperature and humidity. *Aloe arborescens* is propagated vegetatively by shoots appearing in the lower part of the plant. Seedlings rooting takes place in pots using a peat substrate and lasts about a year. After that, plants are planted into the ground.

The plant material—fresh leaves—is collected manually by cutting off the whole plant; therefore, the harvest means the liquidation of the plantation lot. Since the cultivation engages lots of plants at different age stages, the harvest of plant material of certain age could be throughout the year. The age of the crop is counted from the time of planting seedlings in the ground. 

The plantation care treatment is limited to watering and weeding. As succulents, *Aloe* plants are watered moderately, using laboratory-controlled water. Fertilization is controlled, with mineral or organic fertilizers applied in minimal necessary amounts according to the needs and recommendations developed by relevant institutions, based on the results of analyses of soil samples. The risk of contamination is minimal as no pesticides, fungicides, herbicides, or fumigants are used during cultivation. Moreover, the type of plantation (monoculture), cultivation care treatments, and harvest also minimize risks of other contamination types.

### 4.3. Standards and Reagents

Standard substances—aloin A and aloenin A were supplied by PhytoLab. Methanol, Arnov’s reagent, ammonium acetate was supplied by Chempur (Piekary Slaskie, Poland), and methanol HPLC grade by J.T. Baker. Ethanol 96%, formic acid (98–100%), sodium hydroxide, sodium chloride, sodium carbonate, acetate buffer concentrate pH 4.5, and copper (II) chloride 2 hydrate were of analytical grade and were provided by Avantor (Gliwice, Poland), while 2,2-diphenyl-2-picrylhydrazine (DPPH), vitamin C/BHA, neocuproine, Folin-*Ciocâlteu* reagent, and 2,2′-azino-bis(3-ethylbenzothiazoline-6-sulfonic acid (ABTS) were provided by Sigma Aldrich (Schnelldorf, Germany). High-quality pure water was obtained by applying USFT-801 water purification system (Burlington, VT, USA) and Elix SA 67120 millipore purification system (Molsheim, France).

### 4.4. Lyophilization

A pulp was obtained from the leaves by leaf skin separation. It was stored at −20 °C in a freezer for 24 h, and then frozen pulp was lyophilized using a freeze-dryer HetoPowerDry PL3000 Freeze Dryer (Thermo Fisher Scientific, Waltham, MA, USA). The process lasted for 48 h at a temperature of −50 °C under vacuum conditions. Lyophilisates were stored in the conditions of the minimalized humidity and light access.

### 4.5. Extracts Preparation

The extracts were prepared from the lyophilized leaf gel of particular age plants. Then, 100 mg of the lyophilized milled gel were extracted with ethanol 96% (*v*/*v*) or water with the use of ultrasounds. After 30 min, the supernatants were decanted, and the lyophilisates were extracted twice more under the mentioned conditions. Finally, the supernatants collected for each lyophilisate were mixed together, filtered through syringe filters, and concentrated to dryness by applying rotary evaporation. The residues were dissolved in 1 mL of ethanol 96% (*v*/*v*) or water to obtain a 100 mg/mL concentration.

### 4.6. Chromatographic Analysis

#### 4.6.1. Preparation of the Standard Solutions for HPLC Analyses

Two substances were used as standards—aloin A, aloenin A. Their presence in the studied extract was confirmed using mentioned gradient HPLC-DAD method. After setting a baseline, injections of 20 µL volume of extracts were made. Each compound’s identity was confirmed by comparing the retention times of the obtained peaks with the standards. For the purpose of quantitative research, calibration curves were prepared.

#### 4.6.2. Preparation of Extract Solutions for HPLC Analyses

Stock extract (100 mg of lyophilisate in 1 mL of ethanol) was filtered through 0.2 µm syringe filters. Then, 50 µL of the filtered extract was diluted with ethanol up to a 1 mL volume, and the prepared solution was injected. The concentrations of standard compounds were calculated on the basis of the obtained HPLC chromatograms and standard curves.

#### 4.6.3. HPLC Analysis

The RP-HPLC-DAD method was applied to confirm the identity of the material. For this purpose, two standard substances were used as standards—aloin A and aloenin A. In the study, the HPLC Shimadzu Prominence-*i* LC-2030C equipped with DAD detector was used (Shimadzu, Tokyo, Japan). The software was LabSolution DB/CS version 6.50 (Shimadzu, Tokyo, Japan). The stationary phase was LiChrospher 100 RP-18e (250 mm × 4.6 mm, 5 µm) HPLC column (Merck, Warsaw, Poland), the mobile phase comprised of methanol (phase A) and water (phase B) in a gradient (Table S2, Supplementary Materials) the injection volume was 20 µL, and a flow rate was 1 mL/min. The detection was performed at the wavelength λ_max_ = 295 nm.

#### 4.6.4. HPLC-MS/MS Analysis

HPLC-MS/MS analysis was performed using the Shimadzu Nexera coupled to LC-MS 8030 triple quadrupole mass spectrometer (Shimadzu, Tokyo, Japan) with a Kinetex C18 column (100 mm × 2.1 mm, 2.6 µm) (Phenomenex, Torrance, CA, USA). The mobile phase consisted of 0.1% formic acid in methanol (phase A) and water (phase B). Compounds were eluted into the ESI ion source at the flow rate of 0.35 mL/min. The MS was programmed to carry out a full scan over a range of *m*/*z* 100–1000 (MS1) and *m*/*z* 100–500 (MS2) in both positive and negative modes. Electrospray needle voltage was maintained at 4.5 kV. MS interface was adjusted with the following parameters: desolvation line 250 °C, heat block temperature 400 °C, interface temperature 350 °C, nitrogen was used as the drying gas and as the nebulizing gas with flow rates of 15 and 2 L/min, respectively. Compounds were characterized by their MS/MS spectra.

### 4.7. Phenolic Compounds Determination

#### 4.7.1. The Sum of Polyphenolic Compounds

The method is based on a color reaction of the Folin-*Ciocâlteu* reagent with the contained phenolic groups in the presence of sodium carbonate. Therefore, 1 mL of the extract was vortexed with 1 mL of Folin-*Ciocâlteu* reagent for 3 s, then 1 mL of sodium carbonate and 7 mL of water were added and mixed again by vortex for 3 s. After 90 min of incubation in the dark, the resulting colored complex was determined spectrophotometrically at wavelength 765 nm in the Metertech SP-830 spectrophotometer (Metertech, Taipei, Taiwan). In order to determine the content of compounds reactive with the Folin-*Ciocâlteu* reagent, a standard curve for gallic acid was established. Then, using the determined curve, it was converted to the content in 1 g of the dry mass of the extract.

#### 4.7.2. Phenolic Acids

The presence of phenolic acid compounds was determined by the colorimetric method described in Polish Pharmacopoeia VI [46]. The study was performed by adding to 5 mL of distilled water in a volumetric flask, analyzed extract, 0.5 M solution of hydrochloric acid, Arnov’s reagent, and sodium hydroxide in a volume of 1 mL each. After diluting with distilled water to 10 mL volume, spectroscopy measurement was performed at the wavelength of 490 nm. 

The content of phenolic acids was converted to caffeic acid on the basis of on the drawn standard curve and expressed in mg per 1 g of dry mass. 

### 4.8. Antioxidant Activity Determination

#### 4.8.1. CUPRAC Method

The extract’s antioxidant properties were determined by using CUPRAC (cupric ion reducing antioxidant capacity) assay, which was performed according to Apak et al. with modifications [47]. The study is based on the reduction of the complex of neocuproine and cupric ion (II), which results in the change of the sample’s color from bluish to yellow. The CUPRAC reagent consists of equal volumes of 7.5 mM ethanolic 96% (*v*/*v*) solution of neocuproine, 10 mM solution of copper chloride (II), and ammonium acetate buffer of Ph = 7.0. The assay was performed by mixing 50 µL of aloe leaf gel extract with 150 µL of CUPRAC reagent. The solution was shaken and incubated in the dark at room temperature for 30 min. After the incubation, absorbance was measured at the wavelength of λ = 450 nm at Multiskan GO plate reader (Thermo Fisher Scientific, Waltham, MA, USA). As a standard, vitamin C was used. The results were presented as the IC_50_ (inhibitory concentration), which means the minimum concentration of the extract, which reduces the initial, oxidized form of the complex by 50%.

#### 4.8.2. DPPH Method

Determination of activity against DPPH radicals was performed on the basis of the method of Hatano et al. with Amarowicz modifications [48]. The principle of the method was based on the spectrophotometric (Metertech SP-830, Taipei, Taiwan) measurement of the color of the reaction mixture, in which, depending on the antioxidant capacity of the tested extract, free radicals DPPH (1,1-diphenyl-2-pyrrylhydrazyl) were swept away. The absorbance measurement was made at 517 nm after incubation for 30 min at room temperature and protected from light. The calculated percentage of the sweep was put into the standard curve for the Trolox.

#### 4.8.3. ABTS Method

Determination of activity against ABTS cation radicals was performed according to the method described by Re et al. [49]. The test solution was introduced into the reaction medium containing the previously generated cation radical ABTS [2,2′-azinobis (3-ethylbenzothiazoline-6-sulfonate)]. The presence of antioxidants caused the reaction mixture to change color. At the same time, in order to prepare the calibration curve, the absorbance was measured in parallel at the same wavelength of 735 nm of samples containing appropriate concentrations of the reference substance—Trolox, and the results were expressed in its equivalents.

### 4.9. Statistical Analysis

The results were analyzed using Statistica software (version 13.0, Stat Soft Inc., Tulsa, OK, USA). An ANOVA test was used to compare results of the measurements in the studied extracts obtained from the plant at different age. The differences were considered to be significant when *p* ≤ 0.05.

## 5. Conclusions

As a result of the conducted research, we proved that the differences in the content of anthranoid compounds and phenyl pyrone derivatives—aloin A and aloenin A—are significant in lyophilisates obtained from *Aloe arborescens* leaves of different ages (one- to four-year-old). The developed chromatographic method (HPLC-DAD) can be recommended as suitable for the standardization of lyophilisates for the content of aloin A and aloenin A.

The spectrophotometric determination of the sum of polyphenols and phenolic acids revealed the differences in the content of these groups of compounds in lyophilisates obtained from leaves of different ages.

All tested lyophilisates exhibited an antioxidant effect, which is worth emphasizing; the technique for determining the potential should depend on the group of secondary metabolites for which we want to monitor the antioxidant effect.

## Data Availability

Not applicable.

## Supplementary Materials

The following are available online, Table S1. Validation parameters of HPLC-DAD method, Figure S1. Mass spectra for aloin A, Figure S2. Mass spectra for aloenin A, Table S2. HPLC-DAD method gradient. Mobile phase consists of methanol—Phase A and water—Phase B, Table S3. The results of statistical analysis (ANOVA test).

## Sample Availability

Samples of the compounds are available from the authors.
