# Supplementary material for: Analysis of the Composition of Lyophilisates Obtained from Aloe arborescens Gel of Leaves of Different Ages from Controlled Crops
Source: Molecules. 2021 May 27;26(11):3204. doi: 10.3390/molecules26113204 (PMC8198272; doi:10.3390/molecules26113204)
Supplement: Supplementary file 1 [file molecules-26-03204-s001.zip › molecules-1185065-supplementary.pdf]

# Supplementary materials

## Analysis of the composition of lyophilisates obtained from *Aloe arborescens* gel of leaves of different ages from controlled crops

**Kamil Pawłowicz, Dominika Ludowicz, Marta Karaźniewicz-Łada, Kamil Wdowiak and Judyta Cielecka-Piontek**

**Table S1.** Validation parametersof HPLC-DAD method.

| **Parameter** | **Aloin A** | **Aloenin A** |
|---|---|---|
| Retention time – $R_T$ (min) | 16.46 | 12.68 |
| Correlation coefficient – r | 0.9997 | 0.9998 |
| Range of linearity (mg/mL) | 0.0044–0.044 | 0.013–0.13 |
| LOD(mg/mL) | 0.1981 | 0.0917 |
| LOQ (mg/mL) | 0.6003 | 0.2780 |
| Slope (a ± $S_a$) | 26231752.21 ± 1574687.52 | 25840498.78 ± 718467.9855 |
| Intercept (b ± $S_b$) | 130541.2931 ± 44364.84 | −18415.9487 ± 54327.93115 |

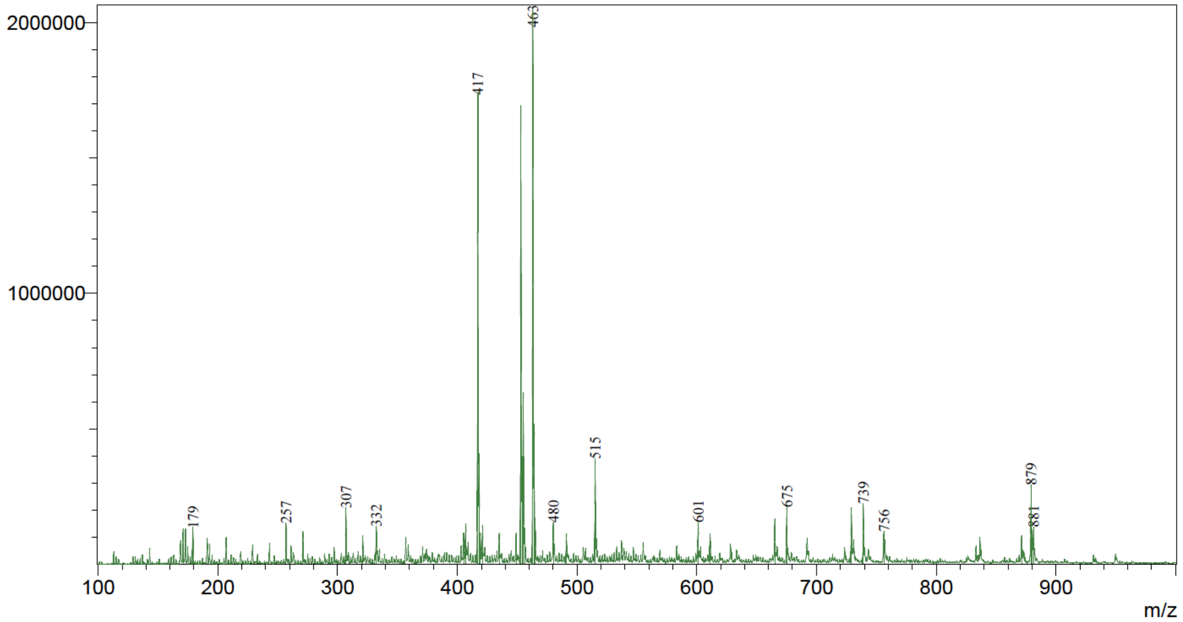


**Figure S1.** Mass spectra for aloin A.

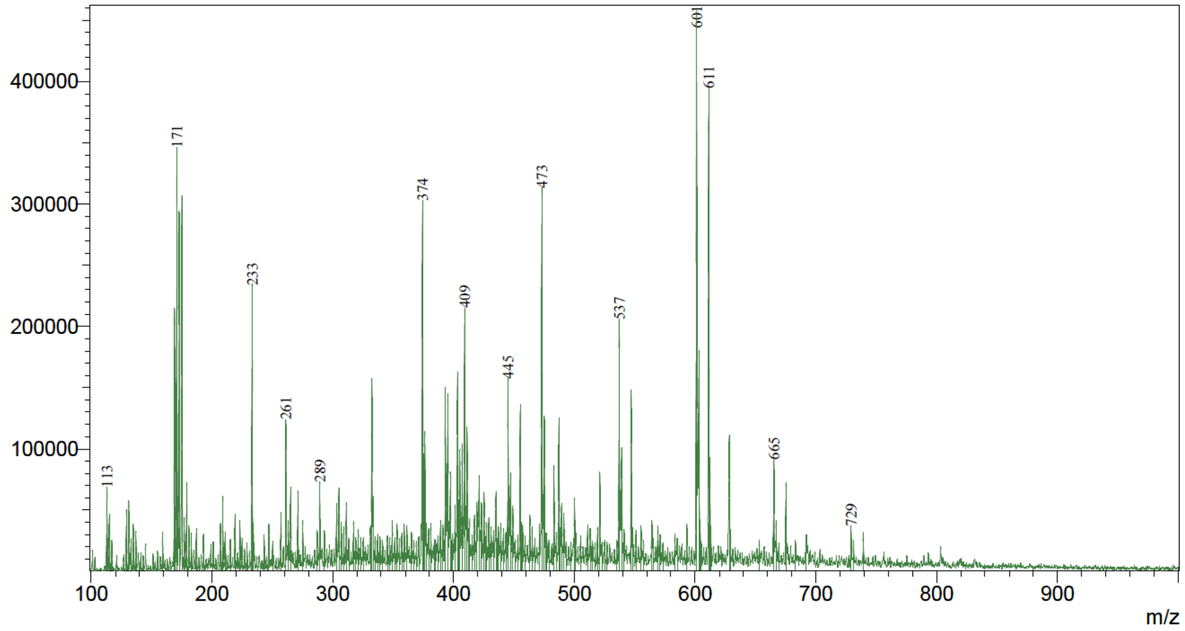


**Figure S2.** Mass spectra for aloenin A.

The HPLC-DAD method developed for the purpose of aloin A and aloenin A determinations in studied extracts, was conducted according to the following conditions of the mobile phase composition.

**Table S2.** HPLC-DAD method gradient. Mobile phase consists of methanol – Phase A and water – Phase B.

| Time (min) | Phase A concentration (%) | Phase B concentration (%) |
|---|---|---|
| 0.0–13.99 | 35 | 65 |
| 14.00–17.00 | 95 | 5 |
| 17.01–20.0 | 35 | 65 |

**Table S3.** The results of statistical analysis (ANOVA test).

| Assay | Type of extract | *p* value |
|---|---|---|
| The sum of polyphenolic compounds | water | 0.0156 |
| | ethanol | 0.0237 |
| Phenolic acids | water | 0.0237 |
| | ethanol | 0.0216 |
| CUPRAC | ethanol | 0.0156 |
| DPPH | water | 0.0156 |
| | ethanol | 0.0473 |
| ABTS | water | 0.0245 |
| | ethanol | 0.0324 |
